# Analysis of Five-Year Trend of Malaria at Bichena Primary Hospital, Amhara Region, Ethiopia

**DOI:** 10.1155/2021/6699373

**Published:** 2021-01-28

**Authors:** Awoke Minwuyelet, Yibeltal Aschale

**Affiliations:** ^1^Bichena Primary Hospital, Amhara Region, Ethiopia; ^2^Department of Medical Laboratory Sciences, College of Health Sciences, Debre Markos University, Debre Markos, Ethiopia

## Abstract

**Background:**

Malaria is a life-threating infectious diseases caused by protozoan parasite of the genus *Plasmodium.* The WHO African region bears the largest burden of malaria morbidity and mortality every year. Prevention and control activity of malaria in Ethiopia is implemented as guided by a national strategic plan to decrease malaria burden. This study is aimed at assessing the five-year trend of malaria at Bichena Primary Hospital.

**Method:**

A retrospective study was conducted at Bichena Primary Hospital to assess the five-year (2015-2019) trend of malaria by reviewing blood film reports from a laboratory logbook.

**Result:**

In a five-year period, 9182 blood films were requested for malaria diagnosis of whom 53.8% were males and 41% were in the age group 15-29. The overall prevalence of malaria was 9.28% (*n* = 852), *P. falciparum* being the dominant malaria species. The highest peaks of total malaria cases were observed in 2016 and in December, and the lowest peaks were observed in 2018 and March (mean annual case 170.4; mean monthly case 14.2), and there was a statistically significant year and monthly variation of malaria cases (*P* < 0.001). Malaria was reported in both sexes and all age groups; of which, males and the age group 15-29 years old consist the highest number of malaria cases (*P* < 0.001).

**Conclusion:**

Malaria remains an important public health problem in the study area, and a significant fluctuation was noticed in a five-year period, *P. falciparum* being slightly the dominant malaria species. Successive efforts are still required to reduce malaria burden to a level that has no longer public health effect.

## 1. Background

Malaria is a life-threating parasitic diseases caused by protozoan parasites of the genus *Plasmodium* and transmitted by female anopheles mosquitoes. It remains a serious global public health problem causing significant morbidity and mortality [1–3]. Five main species of *Plasmodium*, namely, *Plasmodium falciparum*, *Plasmodium vivax*, *Plasmodium malariae*, *Plasmodium ovale*, and *Plasmodium knowlesi*, are able to infect human, of which *P. falciparum* is the most pathogenic species and *P. vivax* also poses a great risk in human [2–4]. Malaria is the leading cause of death in children under the age of 5 years and pregnant women in developing countries [5, 6]. Children with severe malaria frequently develop severe anemia, respiratory distress, and cerebral malaria. Multiorgan failure is also common in adult [7].

Globally, malaria is distributed mainly in three WHO regions, namely, African region, Southeast Asian region, and Eastern Mediterranean region. The WHO African region still bears the largest burden of malaria morbidity and mortality every year [8, 9]. In 2018, an estimated 228 million malaria cases and 405,000 million deaths were recorded worldwide of which 93% (213 million cases) were in WHO African region followed by WHO Southeast Asian region (3.4%) and Eastern Mediterranean region (2.1%). About 272,000 (67%) malaria deaths were estimated to be in children aged less than 5 years old, and 3.3% of all malaria cases were caused by *Plasmodium vivax* in all age groups [9].

The global malaria death rates have declined from 2010 to 2018 by 30.7% (585,000 in 2010 to 405,000 in 2018) and incidence rate (number of cases per 1000 population) of malaria also dropped from 71 in 2010 to 57 in 2018. But, from 2014 to 2018, the rate of change slowed dramatically, reduced from 60 in 2013 to 57 in 2014 and remained unchanged up to 2018. In WHO African region, malaria case incidence level declined by 22% (from 294 in 2010 to 229 in 2018) [9]. This might be an indication of the presence of undetectable parasitemia level and/or misdiagnosis due to lack of more sensitive diagnostic tools in the global effort of malaria elimination [10]. This might be the reason for sustained malaria transmission within population irrespective of active case detection and effective treatment [11].

Malaria is the leading public health problem in Ethiopia. There are about 565 malaria risk districts in Ethiopia (out of 845 total districts), with an estimated at-risk population of 60 million people according to the new stratification [1, 12]. Malaria transmission pattern is unstable and seasonal depending on altitude and rainfall [13]. Peak transmission occurs from September to December in most parts of Ethiopia, following the main rainy season (June to August) and second minor transmission occurs from April to June after a short rainy season (February to March). Since malaria transmission often overlaps with the planting and harvesting season and the majority of malaria burden is among working adults in rural areas, there is heavy economic burden in Ethiopia [12, 14, 15]. Between June 2016 and July 2017, there were 1,530,739 confirmed malaria cases and 356 deaths in all age groups; of which, 69.2% (1,059,847 cases) were due to *P. falciparum* and 30.8% (470,892 cases) were due to *P. vivax* [12].

Nowadays, prevention and control activity of malaria in Ethiopia is implemented as guided by a national strategic plan to ultimately decrease malaria burden to a level that has no longer public health effect. It incorporates four major intervention strategies: early case detection and rapid treatment, vector control via use of indoor residual spraying (IRS), use of long-lasting insecticide-treated mosquito nets, and environmental management [3, 16, 17]. Therefore, the present study is aimed at assessing the five-year back trend of malaria in terms of variation in years, months, and malaria species. Considering these results, stakeholders might give focus on prevention and control of malaria to scale up its elimination in Ethiopia and across malaria-endemic countries.

## 2. Methods

### 2.1. Study Area

The study was conducted at Bichena Primary Hospital located in Bichena Administrative Town, Amhara Region. The town is located in West Central Ethiopia 273 km far from Addis Ababa, capital of Ethiopia, and 225 km far from Bahir Dar, capital of Amhara Regional State, on the hillside overlooking the Abay River. It has a latitude and longitude of 10°27′N 38°12′E and an elevation of 2541 meters above sea level [18]. Bichena Primary Hospital is one of the hospitals found in the region. The hospital renders service for four surrounding districts, namely, Enemay, Enarj Enawga, Debay Tilatgen, and Shebel Berenta. Three out of 38 kebeles in Enemay, three out of 28 kebels in Enarj Enawga, seven out of 21 kebels in Shebel Berenta, and two out of 20 kebels in Debay Tilatgen are malaria endemic (personal communication).

### 2.2. Study Design and Sampling Method

A retrospective study was conducted to assess the five-year (2015-2019) trend of malaria by reviewing blood film reports at Bichena Primary Hospital laboratory logbook. The whole blood films examined over five years in the hospital and fulfilled the inclusion criteria were recorded and analyzed.

### 2.3. Data Collection

Five-year (2015-2019) data regarding malaria were obtained from Bichena Primary Hospital laboratory logbook. In this hospital, a well-prepared and well-stained blood film was used to confirm malaria parasite as recommended by the World Health Organization (WHO) [19]. Both microscopically confirmed positive and negative results were collected by experienced laboratory personnel. Data regarding the patient's age, sex, date, month, and year of examination, blood film result, *Plasmodium* species, and parasite load were collected using a check list prepared for this study. Any incomplete sociodemographic and malaria data records were excluded.

### 2.4. Data Management and Analysis

Data were extracted from laboratory logbooks using well-prepared checklist, entered into SPSS 20 software, checked for completeness, and analyzed accordingly. Descriptive statistics (frequency, percentage, mean, and range) were used to present the data and to evaluate malaria trends over the years and months. A chi-square test was used to describe association of variables such as sex, age, month, year, and parasite load with malaria cases. A statistically significant association was declared at *P* value of <0.05.

## 3. Results

### 3.1. Overall Prevalence and Annual Trend of Malaria

In a five-year period (2015-2019), 9182 blood films were requested for malaria diagnosis at Bichena Primary Hospital of whom 4938 (53.8%) were males, 41% were in the age group 15-29 years old, and 60.9% of patients were diagnosed at outpatient department. The overall malaria prevalence was 9.28% (852/9182). The prevalence of *P. falciparum*, *P. vivax*, and mixed infections was 4.75% (*n* = 436), 3.88% (*n* = 356), and 0.65% (*n* = 60), respectively. Malaria cases occurred throughout the year in spite of variation over a five-year period. The prevalence ranged from 4.02% in 2018 to 18.99% in 2019, and there was a statistically significant year variation (*P* < 0.001). *Plasmodium falciparum* prevalence ranged from 1.69% in 2017 to 6.81% in 2016 and *P. vivax* prevalence ranged from 1.65% in 2018 to 10.0% in 2019 (Table 1).

**Figure 1:**
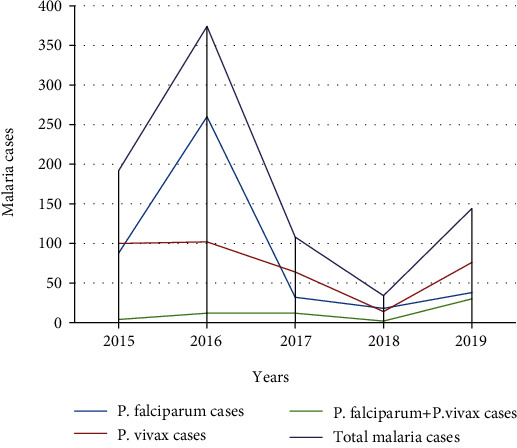
Species trend of malaria at Bichena Primary Hospital from 2015 to 2019.

The mean annual case was 170.4 (range 34-374). The highest peaks of total malaria cases were observed in 2016, and the lowest peaks were observed in 2018. Similarly, the highest and lowest peaks of *P. falciparum* and *P. vivax* cases were observed in 2016 and 2018, respectively (Figure 1).

### 3.2. Malaria Parasite Density and Relative Proportion

*Plasmodium falciparum*, *P. vivax*, and mixed infections accounted 51.2% (436/852), 41.8% (356/852), and 7.0% (60/852) of the total malaria cases, respectively. There was a statistically significant difference in parasite density and proportion among *Plasmodium* species. About 57.2% of malaria cases have a parasite density of 1000-9999 parasites/*μ*L of blood (Table 2).

### 3.3. Average Monthly Variation of Total Malaria Cases and Species

**Figure 2:**
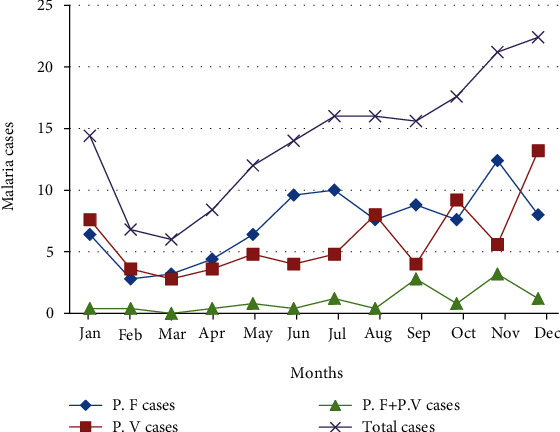
Average monthly variation of total malaria cases and species from 2015 to 2019. P. F: *Plasmodium falciparum*; P. V: *Plasmodium vivax.*

Malaria cases were occurred throughout the months in spite of variation in a five-year period. The prevalence varied among different months ranging from 4.12% to 12.23%, and there was a statistically significant average monthly variation of malaria cases (*χ*^2^ = 77.8; *P* < 0.001). The mean monthly case was 14.2 (range 6-22.4). Relatively highest peaks of total malaria cases were observed during the months of October, November, and December, and the lowest peaks were observed during the months of February and March. The highest *P. falciparum* cases were observed during the months of November, June, and July, and the least cases were observed in the months of February and March. The highest *P. vivax* cases were observed during the months of August, October, and December, and the least cases were observed during February, March, and April (Figure 2).

### 3.4. Prevalence of Malaria among Sex and Age

According to the five-year record in the study area, malaria was reported in all age groups and the prevalence ranged from 4.88% to 10.95%. The age group 15-29 years old comprised the highest malaria case (412 out of the total 852 malaria cases), and the age group ≥ 60 years old consisted the least malaria case (36 out of the total 852 malaria cases). In all age groups, males were more affected than females (malaria cases ranging from 24 to 288 out of 552 total cases). There was a statistically significant difference in malaria cases among sex (*χ*^2^ = 45.3; *P* < 0.001) and age groups (*χ*^2^ = 43.4; *P* < 0.001) (Table 3).

### 3.5. Number of Malaria Cases among the Wards

According to the record, malaria was reported in all wards even if there was a variation among wards ranging from 8 to 522 out of the total 852 malaria cases. Patients diagnosed at OPD ward accounted the highest total malaria case (522 out of the total 852 malaria cases) followed by patients diagnosed at emergency room (286 out of the total 852 malaria cases). The least malaria cases were diagnosed at ANC ward (8 out of total 852 cases). Higher *P. falciparum* cases were diagnosed in OPD and emergency room than *P. vivax* cases, whereas higher *P. vivax* cases were diagnosed at ANC and children's ward (under 5 years of age) than *P. falciparum* cases. There was a statistically significant difference in malaria cases among wards (*P* < 0.001) (Table 4).

## 4. Discussion

This retrospective study was the first-time study conducted in the hospital since its establishment to investigate the five-year trend of malaria. According to this study, 9182 blood films were requested in a five-year period (2015-2019) to confirm malaria parasites and 9.28% (*n* = 852) were found positive for malaria. This result was lower than similar studies conducted in Metema Hospital (17%), Koladiba Health Center (39.6%), Woreta Town, Amhara Region (32.6%), Dembecha Health Center, Northwest Ethiopia (16.34%), and Adi Arkay Health Center, North Gondar Zone (36.1%) [20–24]. In contrast, this finding was higher than studies conducted in Ataye, North Shoa (8.4%), Felegehiwot Referral Hospital, Bahir Dar (5.0%), and Kombolcha, South Wollo (7.52%) [25–27]. This difference might be due to the difference in altitude, public awareness about prevention of malaria, and the ability of the laboratory professionals to detect malaria parasites correctly.

In the present study, malaria cases were observed throughout the year and there was a significant fluctuation (*P* < 0.001) from year to year. The highest peaks of total malaria cases were observed in 2016 and the lowest peaks were observed in 2018. There was a decreasing trend of malaria prevalence from 10.3% in 2015 to 4.02% in 2018, although a significant rise was seen in 2019. Malaria cases dropped from 2016 to 2019 by 88.2% (374 in 2016 to 44 in 2019). This declined trend might be due to successive nationwide effort made by stakeholders to decrease malaria morbidity and mortality. This result agreed with studies conducted in Adi Arkay Health Center, North Gondar [24], Kombolcha, South Wollo [27], Koladiba, North Gondar [21], and Woreta Town, Amhara Region [22]. In contrast, a study conducted in Ataye revealed a nonfluctuating malaria trend [25]. The possible reason for this contradiction might be due to difference in malaria control and prevention activities implemented by stakeholders and climatic factors such as rainfall and temperature.

This study demonstrated that the prevalence of *P. falciparum*, *P.vivax*, and mixed infections was 4.75%, 3.88%, and 0.65%, respectively, and *P. falciparum* was the predominant species consisting 51.2% of total reported malaria cases, although there was a species fluctuation from year to year and month to month. About 57.2% of malaria cases have a parasite density of 1000-9999 parasites/*μ*L of blood. This is in agreement with studies conducted in Dembia District [28], Adi Arkay Health Center [24], Ataye [25], Woreta Town [22], Metema Hospital [20], Kombolcha, South Wollo [27], Koladiba, North Gondar [21], and national malaria operational plan report [12] which reported that *P. falciparum* was the predominant species.

Malaria cases occurred in all months despite variation. The prevalence varied among different months ranging from 4.12% to 12.23%, and there was a statistically significant monthly variation (*P* < 0.001). Highest peaks of total malaria cases were observed during the months of October, November, and December (major malaria transmission seasons), and the lowest peaks were observed during the months of February and March (dry seasons). *P. falciparum* cases were highest in November and least in February. *P. vivax* cases were highest in December and least in March. This is line with studies conducted in Ataye, North Shoa [25], Dembia District [28], Adi Arkay Health Center [24], and Metema Hospital [20] which revealed that malaria cases were increased from September to December and declined in February and March in a 12-month period. The reason might be due to the fact that mosquito breeding is temperature and rainfall dependent.

In this study, malaria cases were reported in both sexes and all age groups. The age group 15-29 years old comprised the highest malaria case followed by 30-44 years old, and the age group ≥ 60 years old accounted the least malaria cases. In all age groups, males were more affected than females. There was a statistically significant difference in malaria cases among sex and age groups (*P* < 0.001). This is in agreement with studies conducted in Metema Hospital and Dembia District by which malaria was reported in both sexes and all age groups and significantly affected age group of 15-29 [20, 28]. Another study conducted in Koladiba, North Gondar and Kombolcha, South Wollo also revealed that the prevalence of malaria was higher among males than females and all age groups were affected by malaria, the age group 15–44 years old being highly affected and ≥65 years old being less affected [21, 27]. The reason behind dominance of malaria cases in male and in the age group 15-44 years old is due to the facts that males in this age group are involved in outdoor activities and are mobile to malaria-endemic areas seeking temporary employment, whereas females do not perform field activities rather they are cookers and stay at home which might reduce the risk of infection.

## 5. Conclusion

The overall prevalence of malaria was found to be 9.28%. This reveals malaria is still an important public health problem in the study area. A significant annual and monthly fluctuation was observed over a five-year period. Successive efforts against malaria are still required to reduce burden to a level that has no longer public health effect. This finding had contribution to stakeholders to understand the barriers to eliminate malaria, how to take action, and the need for further research.

## Figures and Tables

**Table 1:** Prevalence and annual trend of malaria at Bichena Primary Hospital from 2015 to 2019.

Year	Total number of blood films examined	Total number of malaria cases (%)	*P. falciparum* cases (%)	*P. vivax* cases (%)	Mixed (%)	*χ* ^2^ *P* value
2015	1864	192 (10.30)	88 (4.72)	100 (5.36)	4 (0.21)	145.4 <0.001
2016	3816	374 (9.80)	260 (6.81)	102 (2.67)	12 (0.31)	
2017	1898	108 (5.69)	32 (1.69)	64 (3.37)	12 (0.63)	
2018	846	34 (4.02)	18 (2.13)	14 (1.65)	2 (0.23)	
2019	758	144 (18.99)	38 (5.01)	76 (10.0)	30 (3.95)	
Total	9182	852 (9.28)	436 (4.75)	356 (3.87)	60 (0.65)	

**Table 2:** Malaria parasite density and relative proportion from 2015 to 2019.

Species	Parasite density (parasites/*μ*L)			Total (%)	*χ* ^2^ *P* value
	<1000 (%)	1000-9999 (%)	≥10,000 (%)		
*P. falciparum*	88 (20.2)	222 (50.9)	26 (28.9)	436 (51.2)	44.8 <0.001
*P. vivax*	86 (24.1)	226 (63.5)	44 (12.4)	356 (41.8)	
Mixed	2 (3.3)	39 (65)	19 (31.7)	60 (7.0)	
Total	176 (20.6)	487 (57.2)	189 (22.2)	852 (100)	

**Table 3:** Number of malaria cases among age and sex from 2015 to 2019.

Age in years	Malaria cases by sex			*P* value
	Male cases	Female cases	Total cases	
<5	34	12	46	<0.001
5-14	48	38	86	
15-29	288	124	412	
30-44	114	78	192	
45-59	44	36	80	
≥60	24	12	36	
Total	552	300	852	

**Table 4:** Malaria cases among wards in Bichena Primary Hospital from 2015 to 2019.

Wards	*Plasmodium species* detected				*χ* ^2^	*P* value
	P. F cases	P. V cases	P.F + P.V cases	Total cases		
OPD	246	232	44	522	26.8	<0.001
ANC	0	6	2	8		
Emergency	176	98	12	286		
Under 5	14	20	2	36		
Total	436	356	60	852		

ANC: antenatal care; OPD: outpatient department; P. F: *Plasmodium falciparum*; P. V: *Plasmodium vivax.*

## Data Availability

All data generated or analyzed during this study are included in this article.

## Abbreviations

OPD: Outpatient department
ANC: Antenatal care
WHO: World Health Organization.
